# Neoadjuvant Chemoradiotherapy Using Moderately Hypofractionated Intensity-Modulated Radiotherapy Versus Upfront Surgery for Resectable Pancreatic Cancer: A Retrospective Cohort Study

**DOI:** 10.1245/s10434-025-16956-z

**Published:** 2025-02-01

**Authors:** Kei Yamane, Takayuki Anazawa, Kazuyuki Nagai, Yosuke Kasai, Toshihiko Masui, Aya Izuwa, Koki Kurahashi, Satoshi Ishida, Satoshi Ogiso, Michio Yoshimura, Takahiro Iwai, Junichi Matsubara, Akihisa Fukuda, Hiroyoshi Isoda, Yu Hidaka, Yumiko Ibi, Etsuro Hatano

**Affiliations:** 1 Department of Surgery, Graduate School of Medicine, Kyoto University, Kyoto, Japan; 2 Department of Radiation Oncology and Image-Applied Therapy, Graduate School of Medicine, Kyoto University, Kyoto, Japan; 3 Department of Surgery, Kurashiki Central Hospital, Okayama, Japan; 4 Department of Medical Oncology, Graduate School of Medicine, Kyoto University, Kyoto, Japan; 5 Department of Gastroenterology and Hepatology, Graduate School of Medicine, Kyoto University, Kyoto, Japan; 6 Department of Diagnostic Imaging and Nuclear Medicine, Graduate School of Medicine, Kyoto University, Kyoto, Japan; 7 Department of Biomedical Statistics and Bioinformatics, Graduate School of Medicine, Kyoto University, Kyoto, Japan

## Abstract

**Background:**

The efficacy of neoadjuvant chemoradiotherapy for resectable pancreatic ductal adenocarcinoma (R-PDAC) remains unclear. This study was designed to evaluate neoadjuvant chemoradiotherapy by using intensity-modulated radiotherapy (NAC-IMRT) for R-PDAC compared with upfront surgery (UpS).

**Methods:**

Among 198 patients with R-PDAC who were indicated for resection between 2013 and 2021, 130 were included in this study after excluding patients who underwent neoadjuvant chemotherapy and did not meet the NAC-IMRT criteria (Eligible set). NAC-IMRT was planned for 58 patients, and UpS was planned for 72 patients. Additionally, in 105 patients who could undergo the planned treatment (As-treated set), the surgical, pathological, and oncological outcomes were evaluated.

**Results:**

In the Eligible set, median overall survival (OS) was 50.5 months with NAC-IMRT and 34.7 months with UpS and progression-free survival was 20.4 months with NAC-IMRT and 13.9 months with UpS. In the As-treated set, OS was longer in the NAC-IMRT group (66.7 months vs. 34.7 months, *p* = 0.007). On multivariate analysis, NAC-IMRT was identified as an independent factor for better OS (hazard ratio 0.617, 95% confidence interval 0.382–0.995, *p* = 0.047, in the Eligible set). The incidence of postoperative complications did not show a difference between the two groups, and NAC-IMRT suppressed local tumor invasion, including lymphatic, venous, perineural invasion, and lymph node metastases.

**Conclusions:**

NAC-IMRT may offer superior survival outcomes and manageable toxicity in R-PDAC patients compared with upfront surgery. This study supports the efficacy and safety of NAC-IMRT and recommends its consideration in R-PDAC treatment protocols.

**Supplementary Information:**

The online version contains supplementary material available at 10.1245/s10434-025-16956-z.

Pancreatic ductal adenocarcinoma without metastases is classified into “Resectable (R-PDAC)”, “Borderline Resectable (BR-PDAC)”, and “Locally Advanced (LA-PDAC)” according to the tumor attachment and invasion against main vessels, such as superior mesenteric artery, portal vein, common hepatic artery, and celiac trunk.^4^ Among these, R-PDAC is recognized as a type of pancreatic cancer that is more suitable for radical resection, and upfront surgery followed by adjuvant chemotherapy has traditionally been the mainstream treatment. In recent years, the attention of surgeons and clinicians has shifted to neoadjuvant therapy. The benefits of neoadjuvant therapy include eliminating micrometastases and reducing the size of the primary tumor, both of which may be linked to a lower rate of tumor recurrence.^5–7^ The effectiveness of neoadjuvant chemotherapy (NAC) or chemoradiotherapy (NAC-RT) has been demonstrated in numerous clinical trials and retrospective studies, leading to the recommendation of neoadjuvant therapy for BR-PDAC in the National Comprehensive Cancer Network (NCCN) guidelines.^8–11^ However, the application, optimal modality, and regimen of neoadjuvant therapy for R-PDAC remain controversial.

Recent technological innovations in high-precision radiotherapy have made it possible to increase the dose to the local area and improve treatment outcomes for pancreatic cancer.^12^ Intensity-modulated radiotherapy (IMRT) for pancreatic cancer allows for an increased target dose while maintaining a low dose for radiosensitive organs, such as the stomach and duodenum, which change shape daily owing to peristalsis.^13^ We have initiated moderately hypofractionated IMRT for PDAC, reporting that it has enabled dose escalation without increasing gastrointestinal toxicity.^13–15^ Furthermore, we applied this technique to the neoadjuvant treatment for R-PDAC and BR-PDAC and reported good R0 rate and surgical and survival outcomes.^16,17^ However, because these trials were conducted as a single-arm study, it did not clarify whether short-term and oncological outcomes were improved compared with the upfront surgery approach. Intensity-modulated radiotherapy could be a promising option for neoadjuvant therapy. This study was designed to evaluate the efficacy and safety of neoadjuvant chemoradiotherapy using IMRT (NAC-IMRT) for R-PDAC, comparing the short-term and oncological outcomes between upfront surgery and NAC-IMRT.

## Method

### Study Design

The present study was designed as a retrospective observational study using the prospectively maintained institutional database. The study protocol was approved by the ethics committee of Kyoto University Graduate School and Faculty of Medicine (approval code: R1721-3) and conformed to the provisions of the Declaration of Helsinki. Informed consent was obtained from all patients using the opt-out method.

### Patients

We retrieved the data from patients aged 20 to 79, who were diagnosed with R-PDAC between January 2013 and December 2021 at the Kyoto University pancreatic cancer board composed of hepatobiliary pancreatic surgeons, gastroenterologists, medical oncologists, radiation oncologists, and radiologists. All patients were evaluated by using contrast-enhanced computed tomography (CT) and magnetic resonance imaging with a contrast medium of gadolinium-ethoxybenzyl diethylenetriamine pentaacetic acid (EOB-MRI) and positron emission tomography with 2-deoxy-2-[18F] fluoroD-glucose (FDG-PET). R-PDAC is defined as no tumor contact with the SMV or PV, less than 180° contact or invasion without vein contour irregularity, and clear fat planes around the SMA, CA, and CHA, showing no contact or invasion.^18^ All patients were pathologically diagnosed with adenocarcinoma before surgery by using endoscopic ultrasound-fine needle aspiration or pancreatic juice cytology. Patients scheduled for NAC alone were excluded, and further exclusions were made according to the NAC-IMRT eligibility criteria (Supplementary Table S1). We defined cohorts that met the eligibility criteria for NAC-IMRT as the Eligible set. From the Eligible set, we defined the As-treated set as a group excluding cases in which distant metastasis was found after NAC-IMRT or at the time of surgery, cases in which NAC-IMRT could not be completed, and cases in which PDAC was not diagnosed.

### Neoadjuvant Chemoradiotherapy

NAC-IMRT was performed as previously reported.^17^ Briefly, patients were treated with weekly gemcitabine (GEM) at a dose of 1000 mg/m^2^ for 3 weeks as induction chemotherapy before chemoradiotherapy followed by chemoradiotherapy using IMRT with concurrent GEM (days 1, 8, and 15) at a dose of 1000 mg/m^2^. If severe neutropenia or thrombocytopenia occurred, chemotherapy was paused for 1 week. For IMRT planning, the gross tumor volume (GTV) was delineated as pancreatic tumors and lymph nodes >1 cm in diameter. The clinical target volume (CTV) included the retropancreatic regions between the CA and SMA in addition to the GTV plus a 5-mm margin. The planning target volume (PTV) was set as the CTV with a 5-mm margin in all directions. To reduce the high dose to normal organs, the planning organ at risk volume (PRV) was set as the stomach plus 10-mm and duodenum plus 5-mm margins, and GTV-PRV and PTV-PRV, which were calculated by subtracting the PRV from the GTV and PTV, were created as dose prescriptions. The prescribed dose was 45 Gy as D_50_ (D_xx_: the dose covering xx% of the target structure) of GTV-PRV, 42 Gy as D_95_ of PTV-PRV, and PTV D_98_ ≥ 36 Gy in 15 fractions. Intensity-modulated radiotherapy was performed by using exhale breath-hold methods and daily cone-beam CT for verification using volumetric modulated arc therapy.

### Pancreatectomy and Adjuvant Therapy

In the NAC-IMRT group, patients were reevaluated for the indication of pancreatectomy within 4 weeks after neoadjuvant therapy using CT, EOB-MRI, and FDG-PET. The surgical procedure was selected based on the surgeon's judgment. Adjuvant therapy with S-1 (40 mg, 50 mg, or 60 mg according to body surface area, orally administered twice a day for 28 days followed by a 14-day rest) was initiated 4–8 weeks after surgery and administered for 6 months in both groups.

### Data Collection

The patients’ clinicopathological characteristics included age, sex, body mass index (BMI), tumor characteristics, tumor markers, treatment-related variables, and survival data. Preoperative lymph node metastasis was defined as lymph nodes enlarged to 10 mm or more on CT or MRI, or showing FDG uptake on PET-CT. The definition of R0 resection is the absence of residual cancer cells at the resection margins. The neutrophil-to-lymphocyte ratio (NLR) was defined as the ratio of absolute neutrophil count to lymphocyte count, and the modified Glasgow Prognostic Score (mGPS) was calculated based on a previous study.^19^ The Common Terminology Criteria for Adverse Events (CTCAE) version 5.0 was used to evaluate NAC-IMRT toxicity.^20^ Radiological tumor response was evaluated by comparing tumor size on CT images before and after NAC-IMRT according to the Response Evaluation Criteria in Solid Tumors (RECIST v1.1).^21^ Postoperative complications were recorded based on the International Study Group of Pancreatic Surgery definition or the Clavien-Dindo classification.^22–24^ The pathologic response after NAC-IMRT was graded by using Evans’ classification.^25^ The follow-up data were updated in March 2024.

### The Primary and Secondary Endpoints

The primary endpoint is overall survival (OS), which was defined as the period from the date when the treatment plan was decided at the cancer board to either all-cause mortality or the most recent confirmation of survival. The secondary endpoints were progression-free survival (PFS), which is defined as the period from the cancer board to either disease progression or death from any cause, whichever occurred first, and the rates of surgical complications, R0 resection, and local recurrence.

### Statistical Analyses

Continuous variables are expressed as median (interquartile range [IQR]) and were compared by using the Mann-Whitney *U* test. Categorical variables were expressed as frequencies and proportions and compared by using the chi-square test or Fisher’s exact test, depending on the situation. Overall survival and PFS were estimated by using the Kaplan-Meier method and compared between groups by using the log-rank test. To assess the effect of NAC-IMRT on the primary endpoint OS, we used multivariate analysis with a Cox proportional hazards model. The model was adjusted with 11 patient backgrounds that have been shown in previous studies to factor into the predictive outcomes of patients with pancreatic cancer, including age, sex, BMI, and tumor size.^26–28^ The optimal cutoff values of continuous variables were defined based on median values and previous studies.^28^ Multivariate analysis was performed by using the Cox regression model for variables with *p* < 0.100 in the univariate analysis. All statistical calculations were performed by using JMP Pro (version 17.0; SAS Institute Inc., Cary, NC) or GraphPad Prism version 10.3.1 (GraphPad Software, Boston, MA).

## Results

### Patients

**Fig. 1:**
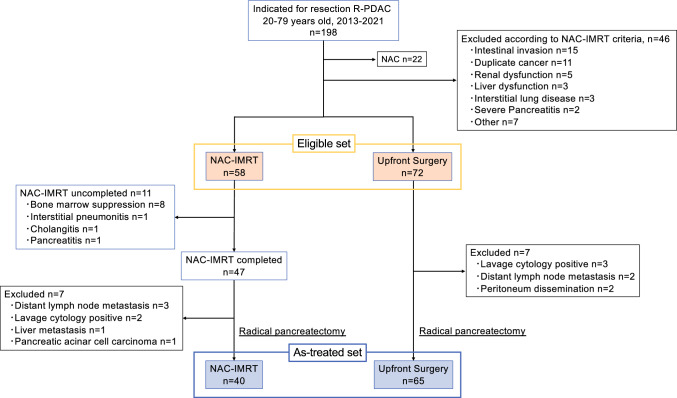
Flowchart of the study

Figure 1 presents the flow diagram of this study. A total of 198 consecutive patients were initially eligible for this study; of these, we excluded 22 patients who were initially planned for NAC and 46 patients who did not meet the NAC-IMRT eligibility criteria. Thus, this study included 130 patients as the Eligible set: 72 UpS group patients and 58 NAC-IMRT group patients. In the UpS group, 7 patients did not undergo curative surgery because of distant metastasis. In the NAC-IMRT group, 11 patients could not complete NAC-IMRT, 6 patients did not receive curative surgery, and 1 patient was diagnosed with pancreatic acinar cell carcinoma. Eventually, 105 patients were included in the as-treated set: 65 patients with UpS and 40 patients with NAC-IMRT. Table 1 shows patient demographics among the Eligible set. Clinical characteristics between the two groups are provided in Supplementary Table S2.

**Table 1:** Patients’ clinical and oncological characteristics at diagnosis among the Eligible set

Variable	Eligible set (n = 130)
Age, years (IQR)	70 (63.8–75)
Sex (male/female)	72 (55.4%) / 58 (44.6%)
BMI, kg/m^2^ (IQR)	21.8 (19.9–23.8)
eGFR, mL/min/1.73 m^2^ (IQR)	76.3 (66.5–89.0)
NAC-IMRT / upfront	58 (44.6%) / 72 (55.4%)
mGPS (0/1,2)	110 (84.6%) / 20 (15.4%)
NLR, median (IQR)	2.19 (1.65–2.87)
Tumor location (head/body, tail)	60 (46.2%) / 70 (53.8%)
Tumor diameter, mm (IQR)	21 (15–25)
Lymph node metastasis	24 (18.5%)
CEA, ng/mL (IQR)	3.5 (1.7–4.9)
CA19-9, U/mL (IQR)	70.1 (25.1–188.1)
Follow-up period, months (IQR)	37.0 (19.9–57.7)

*BMI* body mass index; *CA19-9* carbohydrate antigen19-9; *CEA* carcinoembryonic antigen; *eGFR* estimated glomerular filtration rate; *mGPS* modified Glasgow Prognostic Score; *NLR* neutrophil-to-lymphocyte ratio

### Survival Analysis

**Fig. 2:**
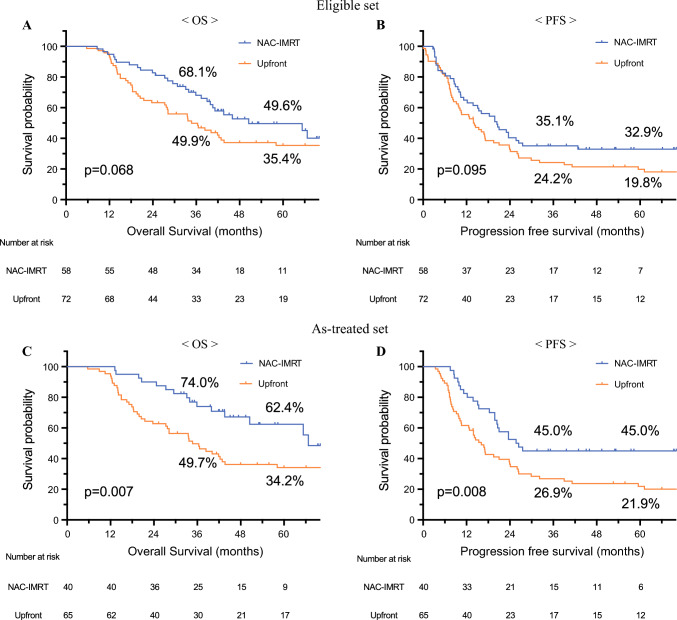
Kaplan-Meier estimates of OS and PFS of the NAC-IMRT and UpS groups in the Eligible set (**A, B**) and in the As-treated set (**C, D**). *OS* overall survival; *PFS* progression-free survival; *NAC-IMRT* neoadjuvant chemoradiotherapy with using intensity-modulated radiation therapy; *UpS* upfront surgery

In the Eligible set, the median OS time was 50.5 months in the NAC-IMRT group and 34.7 months in the UpS group. The survival rates at 3 years were 68.1% versus 49.9% and at 5 years were 49.6% versus 35.4%, respectively (Fig. 2A, *p*= 0.068). The median PFS time was 20.4 months in the NAC-IMRT group, while 13.9 months in the UpS group (Fig. 2B, *p*= 0.095). Although better outcomes were observed in the NAC-IMRT group, no differences were observed between the two groups in terms of OS and PFS. Subgroup analyses based on sex, age, BMI, tumor location, tumor size, CEA levels, and CA19-9 levels demonstrated that NAC-IMRT was favorable in all subgroups (Supplementary Fig. S1). In the As-treated set, the median OS time, and the 3 and 5 years survival rates of the patients in the NAC-IMRT group was significantly better than those in the UpS group (66.7 months, and 74.0% and 62.4% versus 34.7 months, and 49.7% and 34.2% for the NAC-IMRT group vs. UpS group, respectively; *p* = 0.007; Fig. 2C). The median PFS time in the NAC-IMRT group was better than that in the UpS group (26.1 months vs. 16.1 months, *p* = 0.008; Fig. 2D).

### Prognostic Factors for OS

Next, we examined the prognostic factors for OS in both the Eligible set and the As-treated set (Table 2). Eleven clinical and oncological variables were identified as the potential prognostic factors. In the Eligible set, univariate analysis revealed that mGPS = 0 (hazard ratio [HR] 0.566, 95% confidence interval [CI] 0.321–0.998, *p* = 0.049), tumor size >20 mm (HR 1.599, 95% CI 1.010–2.531, *p* = 0.045), lymph node metastasis (HR 2.420, 95% CI 1.431–4.092, *p* = 0.001), and CA19-9 levels >100 U/mL (HR 2.744; 95% CI 1.728–4.356, *p* < 0.001) were significant prognostic factors. Multivariate analysis identified NAC-IMRT (HR 0.617; 95% CI 0.382–0.995, *p* = 0.047), preoperative lymph node metastasis (HR 2.044; 95% CI 1.187–3.519, *p* = 0.009), and CA19-9 >100 U/mL at diagnosis (HR 2.697, 95% CI 1.605–4.532, *p* < 0.001) as significant factors. In the As-treated set, univariate analysis showed that NAC-IMRT (HR 0.458, 95% CI 0.254–0.826, *p* = 0.009), mGPS = 0 (HR 0.420, 95% CI 0.230–0.769, *p* = 0.005), tumor size >20 mm (HR 2.230, 95% CI 1.303–3.817, *p* = 0.004), lymph node metastasis (HR 2.807, 95% CI 1.562–5.044, *p* < 0.001), and CA19-9 levels >100 U/mL (HR 2.889, 95% CI 1.709–4.887, *p* < 0.001) were significant prognostic factors. Multivariate analysis confirmed that NAC-IMRT (HR 0.523, 95% CI 0.290–0.947, *p* = 0.032), mGPS = 0 (HR 0.443, 95% CI 0.238–0.824, *p* = 0.010), preoperative lymph node metastasis (HR 2.214, 95% CI 1.150–3.925, *p* = 0.016), and CA19-9 >100 U/mL at diagnosis (HR 2.336, 95% CI 1.295–4.214, *p* = 0.005) were significant. These results demonstrate that NAC-IMRT was a prognostic factor for patients with R-PDAC who met the eligibility criteria for NAC-IMRT.

**Table 2:** Univariate and multivariate analysis associated with overall survival among the Eligible and As-treated sets (Cox Hazard Model)

	Eligible set				As-treated set			
Variable	Univariate analysis		Multivariate analysis		Univariate analysis		Multivariate analysis	
	HR (95% CI)	*p*	HR (95% CI)	*p*	HR (95% CI)	*p*	HR (95% CI)	*p*
Male	1.147 (0.724–1.816)	0.560	–	–	1.208 (0.718–2.032)	0.476	–	–
Age > 70 yr	1.039 (0.660–1.636)	0.868	–	–	0.945 (0.564–1.583)	0.830	–	–
BMI > 22 kg/m^2^	0.948 (0.599–1.498)	0.818	–	–	1.003 (0.592–1.699)	0.991	–	–
NAC-IMRT	0.647 (0.404–1.037)	0.070	0.617 (0.382–0.995)	0.047*	0.458 (0.254–0.826)	0.009*	0.523 (0.290–0.947)	0.032*
mGPS = 0	0.566 (0.321–0.998)	0.049*	0.612 (0.344–1.089)	0.095	0.420 (0.230–0.769)	0.005*	0.443 (0.238–0.824)	0.010*
NLR > 2.2	0.913 (0.580–1.437)	0.695	–	–	0.917 (0.546–1.540)	0.744	–	–
Pancreas head	0.839 (0.533–1.322)	0.451	–	–	0.729 (0.435–1.222)	0.231	–	–
Tumor size > 20 mm	1.599 (1.010–2.531)	0.045*	1.035 (0.623–1.719)	0.895	2.230 (1.303–3.817)	0.004*	1.503 (0.835–2.708)	0.237
Lymph node metastasis	2.420 (1.431–4.092)	0.001*	2.044 (1.187– 3.519)	0.009*	2.807 (1.562–5.044)	<0.001*	2.124 (1.1–3.925)	0.016*
CEA > 5.0 (ng/mL)	1.570 (0.947–2.605)	0.080	1.487 (0.878– 2.519)	0.140	1.434 (0.805–2.555)	0.221	–	–
CA19-9 > 100 (U/mL)	2.744 (1.728–4.356)	<0.001*	2.697 (1.605–4.532)	<0.001*	2.889 (1.709–4.887)	<0.001*	2.336 (1.295–4.214)	0.005*

*BMI* body mass index; *CA19-9 carbohydrate* antigen19-9; *CEA* carcinoembryonic antigen; *mGPS* modified Glasgow Prognostic Score; *NAC-IMRT* neoadjuvant chemoradiotherapy using intensity-modulated radiotherapy; *NLR* neutrophil-to-lymphocyte ratio

### Preoperative Outcomes and Adverse Effects of NAC-IMRT

**Fig. 3:**
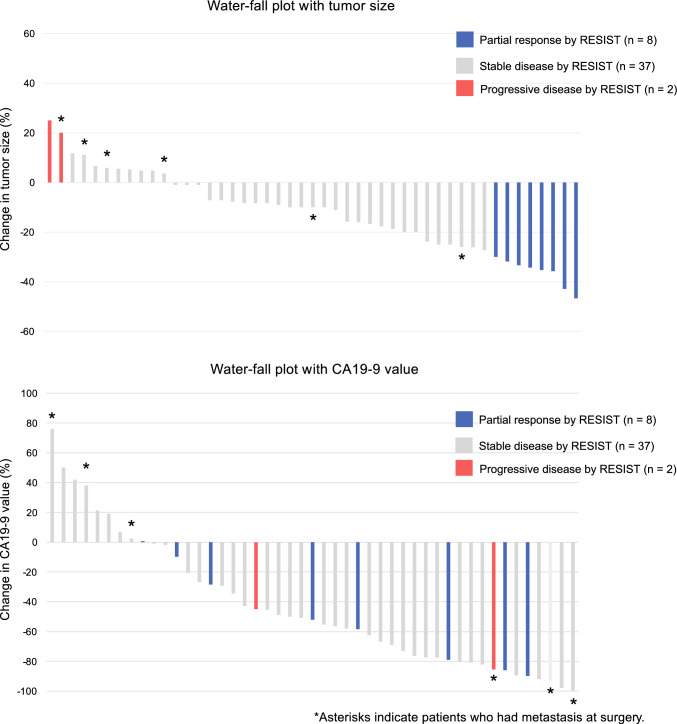
Waterfall plot showing changes in tumor size and CA19-9 levels after NAC-IMRT. Asterisks indicate patients who had metastasis at surgery

Changes in tumor size and CA19-9 levels in patients who completed NAC-IMRT are illustrated using a waterfall plot (Fig. 3). Tumor size decreased in 36 of the 47 cases (76.6%). According to the RECIST classification, there were eight cases (17.0%) with Partial Response, 37 cases (78.7%) with Stable Disease, and two cases (4.3%) with Progressive Disease. CA19-9 levels decreased in 37 of the 47 cases (78.7%). Unresectable factors were found before or during pancreatectomy in 4 of the 11 cases with tumor growth and in 3 of the 9 cases with increased CA19-9 levels. The adverse effects associated with NAC-IMRT are shown in Supplementary Table 3.

### Perioperative and Pathological Outcomes

Table 3 shows the perioperative outcomes and pathological features in As-treated set. In the NAC-IMRT group, minimally invasive surgery was performed in 30.0% of cases, while only 4.6% in the UpS group (*p* < 0.001). The NAC-IMRT group experienced less bleeding (405 mL vs. 760 mL, *p* < 0.001) and shorter hospital stays (21.5 days vs. 25 days, *p* < 0.05). In contrast, the incidence of postoperative complications classified as Clavien-Dindo grade 3a or higher was 15.0% and 18.5%, respectively (*p* = 0.65). In pathological findings, there was no difference in the R0 resection rate between the two groups (95.0% vs. 90.8%, *p* = 0.71). Microscopically, lymphatic, venous, and perineural invasion were observed in 25.0%, 5.0%, and 20.0% of the patients in the NAC-IMRT group, respectively, which were significantly lower than the 49.2%, 20.0%, and 60.0% observed in the UpS group (*p* < 0.05, *p* < 0.001, and *p* < 0.05, respectively). Serosa and retroperitoneum invasion were observed in 35.0% and 37.5% of the NAC-IMRT group, respectively, which were significantly lower than the 64.6% and 63.1% observed in the UpS group (*p* < 0.005 and *p* < 0.05, respectively). The pathological response showed five patients (12.5%) with grade III, and two patients (5.0%) with grade IV according to Evans’ classification. The administration rate of adjuvant therapy was not different between the groups (87.5% vs. 89.2%, *p* = 0.79), and there was no difference in the number of days from surgery to adjuvant therapy (47.5 vs. 41.5 days, *p* = 0.67).

**Table 3:** Perioperative outcomes and pathological features in the As-treated set

Variable		NAC-IMRT (n = 40)	UpS (n = 65)	*p*
Perioperative outcomes	ASA-PS (1,2 / 3,4)	39 (97.5%) / 1 (2.5%)	64 (98.5%) / 1 (1.5%)	1.000
	PD / DP / TP	18 (45.0%) / 22 (55.0%) / 0	35 (53.9%) / 29 (44.6%) / 1 (1.5%)	0.461
	Minimally invasive surgery, n (%)	12 (30.0%)	3 (4.6%)	<0.001*
	Operation time, min (IQR)	443 (380.5–523.8)	479 (334–594.5)	0.697
	Bleeding, mL (IQR)	405 (219.8–615)	760 (362.5–1305)	<0.001*
	Portal vein resection, n (%)	8 (20.0%)	20 (30.8%)	0.23
	Artery resection, n (%)	3 (7.5%)	5 (7.7%)	1.00
	POPF grade B/C, n (%)	6 (15.0%)	8 (12.3%)	0.694
	DGE grade, n (%)	5 (12.5%)	11 (16.9%)	0.590
	Clavien-Dindo classification ≥ IIIa, n (%)	6 (15.0%)	12 (18.5%)	0.648
	Hospital stays, days (IQR)	21.5 (15.3–27.5)	25 (18–34.5)	0.045*
	Adjuvant chemotherapy, n (%)	35 (87.5%)	58 (89.2%)	0.764
	Days from surgery to adjuvant chemotherapy, days (IQR)	47.5 (31–61.3)	41.5 (33.5–56.5)	0.759
	Recurrence, n (%)	22 (55.0%)	51 (78.5%)	0.011*
Pathological features	R0 resection, n (%)	38 (95.0%)	59 (90.8%)	0.707
	Pancreatic cut-end margin positive, n (%)	1 (2.5%)	1 (1.5%)	1.00
	Dissected peripancreatic tissue margin positive, n (%)	1 (2.5%)	5 (7.7%)	0.404
	Tumor diameter, mm (IQR)	21 (14–25)	26 (21.3–35.8)	<0.001*
	Lymph node metastasis, n (%)	10 (25.0%)	32 (49.2%)	0.014*
	Lymphatic invasion, n (%)	2 (5.0%)	13 (20.0%)	0.033*
	Venous invasion, n (%)	8 (20.0%)	39 (60.0%)	<0.001*
	Perineural invasion, n (%)	22 (55.0%)	50 (76.9%)	0.019*
	Serosa invasion, n (%)	14 (35.0%)	42 (64.6%)	0.003*
	Retroperitoneum invasion, n (%)	15 (37.5%)	41 (63.1%)	0.011*
	Evans classification (I, IIa, IIb/ II/ IV)	33 (82.5%) / 5 (12.5%) / 2 (5.0%)	–	–

*ASA-PS* American Society of Anesthesiologists physical status; *DGE* delayed gastric emptying; *DP* distal pancreatectomy; *PD* pancreaticoduodenectomy; *POPF* postoperative pancreatic fistula; *TP* total pancreatectomy; *IQR* interquartile range

### Initial Recurrence Sites and Therapies for Recurrence

Among the As-treated patients, 22 (55.0%) patients developed recurrence during the follow-up period in the NAC-IMRT group and 51 (78.5%) in the UpS group (*p* = 0.011). Table 4 shows the sites of initial recurrence and therapies for recurrence in both the groups. The incidence of initial local recurrence was lower in the NAC-IMRT group (9.1% and 21.6%, *p* = 0.320). The number of patients who received GnP or mFOLFIRINOX was 16 (72.7%) in the NAC-IMRT group and 23 (45.1%) in the UpS group (*p* = 0.041). Metastatectomy was performed in 22.7% and 9.8% of the patients in the NAC-IMRT and UpS groups, respectively (*p* = 0.157). Radiotherapy was administered to 4.6% of patients in the NAC-IMRT (for lung metastasis) and 17.7% in the UpS group (*p* = 0.264).

**Table 4:** Site of initial recurrence and treatments for recurrence

Variable		NAC-IMRT (n = 22)	UpS (n = 51)	*p*
Initial recurrence site (including duplicates)	Liver	9 (40.9%)	17 (33.3%)	0.535
	Lung	9 (40.9%)	9 (17.6%)	0.034*
	Local	2 (9.1%)	11 (21.6%)	0.320
	Peritoneum	4 (18.2%)	19 (37.3%)	0.169
	Lymph node	0 (0%)	8 (15.7%)	0.096
	Remnant pancreas	0 (0%)	5 (9.8%)	0.314
	Other	0 (0%)	2 (3.9%)	1.000
Chemotherapy for recurrence	GnP or mFOLFIRINOX	16 (72.7%)	23 (45.1%)	0.041*
	another regimen	4 (18.1%)	20 (39.2%)	0.106
	BSC or unknown	2 (9.1%)	8 (15.7%)	0.713
Locoregional treatment for recurrence	Metastatectomy	5 (22.7%)	5 (9.8%)	0.157
	Radiotherapy	1 (4.6%)	9 (17.7%)	0.264
	Radiofrequency ablation	1 (4.6%)	0 (0%)	0.301

*GnP* gemcitabine plus nab-paclitaxel; *mFOLFIRINOX* modified fluorouracil, leucovorin, irinotecan, and oxaliplatin

## Discussion

In the present study, we conducted a comprehensive review of patients diagnosed with R-PDAC who met the eligibility criteria for NAC-IMRT. In the As-treated set, where patients completed neoadjuvant therapy and surgery, the survival benefit of NAC-IMRT was statistically demonstrated. However, in the Eligible set, which included cases where neoadjuvant therapy was discontinued or distant metastases appeared, no statistically significant differences in OS or PFS were observed between the NAC-IMRT group and the control group. This suggests that some patients may not derive substantial benefits from NAC-IMRT. Although the tumor backgrounds were expected to be similar between the two groups, the degree of local invasion in the pathological findings was significantly reduced, and significantly fewer regional lymph node metastases were observed in the NAC-IMRT group. Based on the short-term and oncological outcomes demonstrated in this study, the efficacy of NAC-IMRT for R-PDAC is considered acceptable.

The theoretical advantages of neoadjuvant therapy (NAT) for pancreatic cancer are multifaceted. First, an important advantage is that patients can receive chemotherapy while in good systemic condition before surgery, which increases the proportion of patients who undergo chemotherapy.^29^ Some patients cannot receive adjuvant chemotherapy after surgery because of complications or a decline in performance status.^30^ Pancreatic cancer has a poor prognosis with surgery alone; therefore, receiving chemotherapy during the perioperative period may improve the survival time. Next, NAT can shrink the tumor, allowing for a reduction in the extent of resection and the number of organs removed during subsequent surgery, thereby improving the rate of curative resection.^31^ Furthermore, evaluating the clinical response to NAT can help predict the effectiveness and side effects of adjuvant chemotherapy.^32^

NAC-IMRT is considered to be a treatment that has the potential to meet these benefits. First, receiving both chemotherapy and radiotherapy prior to surgery is advantageous as it facilitates a multimodal treatment approach, which is associated with improved survival outcomes in various cancer types including PDAC.^33^ Second, the acceptance rate of postoperative adjuvant chemotherapy was comparable between both groups. This is likely because the postoperative complication rate did not worsen with NAC-IMRT. Adjuvant chemotherapy is an important therapy after pancreatectomy,^34,35^ and its acceptance rate should not be reduced by preoperative treatment. Next, the therapeutic effect of NAC-IMRT on the local area was clarified by the results of this study; lymphatic invasion, venous invasion, neural invasion, peripancreatic invasion, and even regional lymph node metastasis were suppressed by NAC-IMRT. This was also the case in the PREOPANC study and is considered to clearly show the therapeutic effect of preoperative radiotherapy on the local area.^36^ This may also explain why the NAC-IMRT group had less local and remnant pancreatic recurrence.

Several randomized trials have demonstrated the superiority of NAC-RT over upfront surgery for R or BR-PDAC, consistent with the findings of the present study.^36–39^ However, NAC-RT has not been proven superior to NAC in the A021501 or PREOPANC-2 trials. This may be because the A021501 trial did not include chemotherapy with RT, and the PREOPANC-2 trial used a lower radiation dose of 36 Gy in 15 fractions compared to this study. Also, both trials targeted only the tumor and major vessels in contact with the tumor, leaving out prophylactic areas. Recent studies have reported that treating only the area surrounding the primary tumor results in insufficiently prescribed RT doses, leading to a high-risk area for local recurrence. By contrast, the RT regimen in the present study included 15 fractions of concurrent chemotherapy and incorporated the prophylactic area. Further research is needed to evaluate whether our RT protocol is superior to neoadjuvant chemotherapy.

NAC-IMRT allows preoperative evaluation of the effectiveness of neoadjuvant treatment. As shown in Fig. 3, 36.4% of cases with tumor enlargement during NAC-IMRT or 33.3% of cases with an increase in CA19-9 levels could not complete radical resection due to the appearance of distant metastasis. This result suggests that clinical response to NAC-IMRT may be linked to prognosis prediction. Predicting the occurrence of distant metastasis based solely on the rate of change in CA19-9 level is challenging. Patients with high CA19-9 levels or large tumor sizes at the time of pancreatic cancer diagnosis may have undetectable unresectable factors on imaging.^40^ In this study, performing staging laparoscopy before neoadjuvant therapy in such cases may have prevented the inclusion of patients with distant metastases.

The present study had several limitations. First, this was a single-center, nonrandomized trial, and the sample size was small and insufficient to accurately validate the outcomes. Second, the effect of NAC-IMRT on the primary endpoint was evaluated by using multivariate analysis to adjust for confounding factors. However, we were unable to address unmeasured confounding factors. While a randomized controlled trial would be needed to clarify the true treatment effects, it is unrealistic to design such a trial because of the difficulty of standardizing the IMRT treatment in a multicenter study. Third, this study includes patients from 2013 through 2021, with the NAC-IMRT cohort from a more recent period. As shown in Supplementary Fig. S2, GnP or mFOLFIRINOX did not improve OS, but improvements in the management of pancreatic cancer patients other than neoadjuvant therapy and chemotherapy after recurrence may have influenced the prognosis of the NAC-IMRT group. Advancements in imaging techniques may also have influenced patient selection between the two groups.

## Conclusion

NAC-IMRT may provide a survival benefit for patients with R-PDAC owing to the suppression of local tumor invasion and regional lymph node metastasis. By administering chemoradiotherapy preoperatively, patients can undergo multidisciplinary treatment for pancreatic cancer, which ultimately leads to an improved prognosis.

## Supplementary Information

Below is the link to the electronic supplementary material.

Supplementary file1 (DOCX 287 KB)

## References

[1] Kamisawa T, Wood LD, Itoi T, et al. Pancreatic cancer. *Lancet*. 2016;388:73–85. 26830752 10.1016/S0140-6736(16)00141-0

[2] Vincent A, Herman J, Schulick R, et al. Pancreatic cancer. *Lancet*. 2011;378:607–20. 21620466 10.1016/S0140-6736(10)62307-0 PMC3062508

[3] McGuigan Andrew, Kelly Paul, Turkington Richard C, Jones Claire, Coleman Helen G, Stephen McCain R. Pancreatic cancer: a review of clinical diagnosis, epidemiology, treatment and outcomes. *World J Gastroenterol*. 2018;24(43):4846–61. 10.3748/wjg.v24.i43.4846. 30487695 10.3748/wjg.v24.i43.4846 PMC6250924

[4] Isaji S, Mizuno S, Windsor JA, Bassi C, Fernandez-Del Castillo C, Hackert T, et al. International consensus on definition and criteria of borderline resectable pancreatic ductal adenocarcinoma, 2017. *Pancreatology*. 2018;2017(18):2–11. 10.1016/j.pan.2017.11.011 29191513

[5] Zhan HX, Xu JW, Wu D, Wu ZY, Wang L, Hu SY, Zhang GY. Neoadjuvant therapy in pancreatic cancer: a systematic review and meta-analysis of prospective studies. *Cancer Med*. 2017;6:1201–19. 28544758 10.1002/cam4.1071 PMC5463082

[6] Sohal DP, Walsh RM, Ramanathan RK, Khorana AA. Pancreatic adenocarcinoma: treating a systemic disease with systemic therapy. *JNCI J Natl Cancer Inst*. 2014;106(3):dju011–dju011. 10.1093/jnci/dju011. 24563516 10.1093/jnci/dju011

[7] Zou Y, Gao S, Yu X, Zhou T, Xie Y, Guo X, et al. Survival outcomes of neoadjuvant therapy followed by radical resection versus upfront surgery for stage I-III pancreatic ductal adenocarcinoma: a retrospective cohort study. *Int J Surg*. 2023;109:1573–83. 37132194 10.1097/JS9.0000000000000425 PMC10389558

[8] Nagakawa Y, Sahara Y, Hosokawa Y, Murakami Y, Yamaue H, Satoi S, et al. Clinical impact of neoadjuvant chemotherapy and chemoradiotherapy in borderline resectable pancreatic cancer: analysis of 884 patients at facilities specializing in pancreatic surgery. *Ann Surg Oncol*. 2019;26:1629–36. 10.1245/s10434-018-07131-8. 30610555 10.1245/s10434-018-07131-8

[9] Miyasaka Yoshihiro, Ohtsuka Takao, Kimura Ryuichiro, Matsuda Ryota, Mori Yasuhisa, Nakata Kohei, Kakihara Daisuke, Fujimori Nao, Ohno Takamasa, Oda Yoshinao, Nakamura Masafumi. Neoadjuvant chemotherapy with gemcitabine plus nab-paclitaxel for borderline resectable pancreatic cancer potentially improves survival and facilitates surgery. *Ann Surg Oncol*. 2019;26(5):1528–34. 10.1245/s10434-019-07309-8. 30868514 10.1245/s10434-019-07309-8

[10] Versteijne E, van Dam JL, Suker M, Janssen QP, Groothuis K, Akkermans-Vogelaar JM, et al. Neoadjuvant chemoradiotherapy versus upfront surgery for resectable and borderline resectable pancreatic cancer: long-term results of the Dutch Randomized PREOPANC Trial. *J Clin Oncol*. 2022;40:1220–30. 35084987 10.1200/JCO.21.02233

[11] N.C.C. Network, NCCN clinical practice guidelines in oncology (NCCN guidelines); 2024.

[12] Reyngold M, O’Reilly EM, Varghese AM, Fiasconaro M, Zinovoy M, Romesser PB, et al. Association of ablative radiation therapy with survival among patients with inoperable pancreatic cancer. *JAMA Oncol*. 2021;7:735–8. 33704353 10.1001/jamaoncol.2021.0057 PMC7953335

[13] Ogawa A, Yoshimura M, Nakamura M, Adachi T, Iwai T, Ashida R, Mizowaki T. Impact of planning organ at risk volume margins and matching method on late gastrointestinal toxicity in moderately hypofractionated IMRT for locally advanced pancreatic ductal adenocarcinoma. *Radiat Oncol*. 2023;18:103. 37337247 10.1186/s13014-023-02288-3 PMC10280835

[14] Goto Y, Nakamura A, Ashida R, Sakanaka K, Itasaka S, Shibuya K, et al. Clinical evaluation of intensity-modulated radiotherapy for locally advanced pancreatic cancer. *Radiat Oncol*. 2018;13:118. 29940984 10.1186/s13014-018-1063-5 PMC6019294

[15] Iwai T, Yoshimura M, Ashida R, Goto Y, Kishi T, Itasaka S, et al. Hypofractionated intensity-modulated radiotherapy with concurrent chemotherapy for elderly patients with locally advanced pancreatic carcinoma. *Radiat Oncol*. 2020;15:264. 33187523 10.1186/s13014-020-01712-2 PMC7666451

[16] Masui T, Nagai K, Anazawa T, Sato A, Uchida Y, Nakano K, et al. Impact of neoadjuvant intensity-modulated radiation therapy on borderline resectable pancreatic cancer with arterial abutment; a prospective, open-label, phase II study in a single institution. *BMC Cancer*. 2022;22:119. 35093003 10.1186/s12885-022-09244-6 PMC8800301

[17] Masui T, Nagai K, Anazawa T, Kasai Y, Yogo A, Yoshimura M, et al. Safety and efficacy of neoadjuvant chemoradiotherapy with moderately hypofractionated intensity-modulated radiotherapy for resectable pancreatic cancer: a prospective, open-label, phase II study. *Cancer Med*. 2023;12:18611–21. 37649318 10.1002/cam4.6470 PMC10557863

[18] J.P. Society, Classification of pancreatic carcinoma, 4th ed. Kanehara & Co., LTD, 2017

[19] Koike Y, Miki C, Okugawa Y, Yokoe T, Toiyama Y, Tanaka K, et al. Preoperative C-reactive protein as a prognostic and therapeutic marker for colorectal cancer. *J Surg Oncol*. 2008;98:540–4. 18937231 10.1002/jso.21154

[20] Common Terminology Criteria for Adverse Events v5.0 (CTCAE).

[21] Eisenhauer EA, Therasse P, Bogaerts J, Schwartz LH, Sargent D, Ford R, et al. New response evaluation criteria in solid tumours: revised RECIST guideline (version 1.1). *Eur J Cancer*. 2009;45:228–47. 19097774 10.1016/j.ejca.2008.10.026

[22] Bassi C, Marchegiani G, Dervenis C, Sarr M, Abu Hilal M, Adham M, et al. The 2016 update of the international study group (ISGPS) definition and grading of postoperative pancreatic fistula: 11 years after. *Surgery*. 2017;16:584–91. 10.1016/j.surg.2016.11.014 28040257

[23] Wente MN, Bassi C, Dervenis C, Fingerhut A, Gouma DJ, Izbicki JR, et al. Delayed gastric emptying (DGE) after pancreatic surgery: a suggested definition by the international study group of pancreatic surgery (ISGPS). *Surgery*. 2007;142:761–8. 17981197 10.1016/j.surg.2007.05.005

[24] Clavien PA, Barkun J, de Oliveira ML, Vauthey JN, Dindo D, Schulick RD, et al. The Clavien-Dindo classification of surgical complications: five-year experience. *Ann Surg*. 2009;250:187–96. 19638912 10.1097/SLA.0b013e3181b13ca2

[25] Evans DB, Rich TA, Byrd DR, Cleary KR, Connelly JH, Levin B, et al. Preoperative chemoradiation and pancreaticoduodenectomy for adenocarcinoma of the pancreas. *Arch Surg*. 1992;127:1335–9. 1359851 10.1001/archsurg.1992.01420110083017

[26] van Eijck CWF, Mustafa DAM, Vadgama D, de Miranda N, Groot Koerkamp B, van Tienhoven G, et al. Enhanced antitumour immunity following neoadjuvant chemoradiotherapy mediates a favourable prognosis in women with resected pancreatic cancer. *Gut*. 2024;73:311–24. 37709493 10.1136/gutjnl-2023-330480 PMC10850691

[27] Matsumoto I, Murakami Y, Shinzeki M, Asari S, Goto T, Tani M, et al. Proposed preoperative risk factors for early recurrence in patients with resectable pancreatic ductal adenocarcinoma after surgical resection: a multi-center retrospective study. *Pancreatology*. 2015;15:674–80. 26467797 10.1016/j.pan.2015.09.008

[28] Yamada S, Hashimoto D, Yamamoto T, Yamaki S, Oshima K, Murotani K, et al. Reconsideration of the clinical impact of neoadjuvant therapy in resectable and borderline resectable pancreatic cancer: a dual-institution collaborative clinical study. *Pancreatology*. 2024;24:592–9. 38548551 10.1016/j.pan.2024.03.012

[29] Oba A, Ho F, Bao QR, Al-Musawi MH, Schulick RD, Del Chiaro M. Neoadjuvant treatment in pancreatic cancer. *Front Oncol*. 2020;10:245. 32185128 10.3389/fonc.2020.00245 PMC7058791

[30] Springfeld C, Ferrone CR, Katz MHG, Philip PA, Hong TS, Hackert T, et al. Neoadjuvant therapy for pancreatic cancer. *Nat Rev Clin Oncol*. 2023;20:318–37. 36932224 10.1038/s41571-023-00746-1

[31] Kolbeinsson HM, Chandana S, Wright GP, Chung M. Pancreatic cancer: a review of current treatment and novel therapies. *J Invest Surg*. 2023;36:2129884. 36191926 10.1080/08941939.2022.2129884

[32] Dallavalle S, Campagnoli G, Pastena P, Martinino A, Schiliro D, Giovinazzo F. New frontiers in pancreatic cancer management: current treatment options and the emerging role of neoadjuvant therapy. *Medicina (Kaunas)*. 2024;60:1070. 39064499 10.3390/medicina60071070 PMC11278520

[33] Pierobon ES, Capovilla G, Moletta L, De Pasqual AL, Fornasier C, Salvador R, et al. Multimodal treatment of radiation-induced esophageal cancer: results of a case-matched comparative study from a single center. *Int J Surg*. 2022;99:106268. 35183734 10.1016/j.ijsu.2022.106268

[34] Oettle H, Neuhaus P, Hochhaus A, Hartmann JT, Gellert K, Ridwelski K, et al. Adjuvant chemotherapy with gemcitabine and long-term outcomes among patients with resected pancreatic cancer: the CONKO-001 randomized trial. *JAMA*. 2013;310:1473–81. 24104372 10.1001/jama.2013.279201

[35] Uesaka K, Boku N, Fukutomi A, Okamura Y, Konishi M, Matsumoto I, et al. Adjuvant chemotherapy of S-1 versus gemcitabine for resected pancreatic cancer: a phase 3, open-label, randomised, non-inferiority trial (JASPAC 01). *Lancet*. 2016;388:248–57. 27265347 10.1016/S0140-6736(16)30583-9

[36] Versteijne E, Suker M, Groothuis K, Akkermans-Vogelaar JM, Besselink MG, Bonsing BA, et al. Preoperative chemoradiotherapy versus immediate surgery for resectable and borderline resectable pancreatic cancer: results of the Dutch Randomized Phase III PREOPANC Trial. *J Clin Oncol*. 2020;38:1763–73. 32105518 10.1200/JCO.19.02274 PMC8265386

[37] Casadei R, Di Marco M, Ricci C, et al. Neoadjuvant chemoradiotherapy and surgery versus surgery alone in resectable pancreatic cancer: a single-center prospective, randomized, controlled trial which failed to achieve accrual targets. *J Gastrointest Surg*. 2015;19:1802–12. 26224039 10.1007/s11605-015-2890-4

[38] Golcher H, Brunner TB, Witzigmann H, et al. Neoadjuvant chemoradiation therapy with gemcitabine/cisplatin and surgery versus immediate surgery in resectable pancreatic cancer: Results of the first prospective randomized phase II trial. *Strahlenther Onkol*. 2015;191:7–16. 25252602 10.1007/s00066-014-0737-7 PMC4289008

[39] Jang JY, Han Y, Lee H, et al. Oncological benefits of neoadjuvant chemoradiation with gemcitabine versus upfront surgery in patients with borderline resectable pancreatic cancer: a prospective, randomized, open-label, multicenter phase 2/3 trial. *Ann Surg*. 2018;268:215–22. 29462005 10.1097/SLA.0000000000002705

[40] Sakaguchi T, Satoi S, Hashimoto D, Yamamoto T, Yamaki S, Hirooka S, et al. A simple risk score for detecting radiological occult metastasis in patients with resectable or borderline resectable pancreatic ductal adenocarcinoma. *J Hepatobiliary Pancreat Sci*. 2022;29:262–70. 34314568 10.1002/jhbp.1026

