# Supplementary material for: Neoadjuvant Chemoradiotherapy Using Moderately Hypofractionated Intensity-Modulated Radiotherapy Versus Upfront Surgery for Resectable Pancreatic Cancer: A Retrospective Cohort Study
Source: Ann Surg Oncol. 2025 Feb 1;32(5):3603–13. doi: 10.1245/s10434-025-16956-z (PMC11976822; doi:10.1245/s10434-025-16956-z)
Supplement: Supplementary file 1 — Supplementary file1 (DOCX 287 KB) [file 10434_2025_16956_MOESM1_ESM.docx]

**Supplemental Digital Content**

**Neoadjuvant chemoradiotherapy using moderately hypofractionated intensity-modulated radiotherapy versus Upfront surgery for resectable pancreatic cancer: a retrospective cohort study**

**Table S1.** Inclusion and exclusion criteria for NAC-IMRT.

**Table S2.** Patients' clinical and oncological characteristics at diagnosis according to the NAC-IMRT and UpS groups.

**Table S3.** Adverse effects of NAC-IMRT.

**Figure S1.** Hazard Ratios for the Impact of NAC-IMRT on Overall Survival Across Subgroups.

**Figure S2.** Kaplan–Meier estimates of OS comparing recurrent cases in the As-treated set based on the presence or absence of GnP or mFFX therapy.

| Table S1. Inclusion and exclusion criteria for NAC-IMRT |
| --- |
| Inclusion criteria |
| 1. Age ≥20 and <80 years |
| 2. ECOG Performance Status（PS）0 or 1 |
| 3. Histologically confirmed pancreas adenocarcinoma or adenosquamous carcinoma |
| 4. Resectable pancreatic cancer as defined by the Classification of Pancreatic Carcinoma in JAPAN; specifically: No tumor contacts with SMV or PV or less than 180 contact or invasion without occlusion. Clear fat planes around SMA CA, CHA, showing no contact or invasion. |
| 5. Does not show evident invasion into the mucosa of the stomach or intestines. |
| 6. Distant metastasis is not confirmed. |
| 7. The functions of major organs are preserved. |
| a. WBC ≥3,500/mm3 and <12,000/mm3, NEUT ≥2,000/mm3, Hb ≥9.0g/dL, Plt ≥100,000/mm3 |
| b. T-Bil 2.0mg/dL, AST ≤150U/L, ALT ≤150U/L |
| c. Cre 1.2mg/dL, eGFR ≥50mL/min |
| Exclusion criteria |
| 1. Pulmonary fibrosis or interstitial pneumonia. |
| 2. Previous radiation therapy to the upper abdomen. |
| 3. Double cancer |
| 4. Pregnant |
| 5. Judged inappropriate to conduct this study safely. |

| Table S2. Patients' clinical and oncological characteristics at diagnosis according to the NAC-IMRT and UpS groups. | | | |
| --- | --- | --- | --- |
| Variable | NAC-IMRT (n=58) | UpS (n=72) | p value |
| Age, >70 years, n (%) | 32 (55.2%) | 35 (48.6%) | 0.457 |
| Sex, male, n (%) | 31 (53.4%) | 41 (56.9%) | 0.690 |
| BMI >22.0 kg/m2, n (%) | 36 (62.1%) | 41 (56.9%) | 0.555 |
| mGPS: 0, n (%) | 51 (87.9%) | 59 (81.9%) | 0.347 |
| NLR >2.2, n (%) | 36 (62.1%) | 29 (40.2%) | 0.021† |
| Tumor location, head, n (%) | 27 (46.6%) | 33 (45.8%) | 0.930 |
| Tumor diameter >20 mm, n (%) | 26 (44.8%) | 40 (55.6%) | 0.224 |
| Lymph node metastasis, n (%) | 7 (12.1%) | 17 (23.6%) | 0.092 |
| Serum CEA levels >5.0 ng/mL, n (%) | 12 (20.7%) | 19 (26.4%) | 0.449 |
| Serum CA19-9 levels >100 U/mL, n (%) | 23 (39.7%) | 31 (43.1%) | 0.696 |
| Abbreviations: BMI, body mass index; CA19-9, carbohydrate antigen19-9; CEA, carcinoembryonic antigen; mGPS, modified Glasgow Prognostic Score; NLR; neutrophil to lymphocyte ratio | | | |

| Table S3. Adverse effects of NAC-IMRT | | |
| --- | --- | --- |
|  | All | ≥ Grade3 |
| Abdominal pain | 7 (12.1%) | 0 |
| Anemia | 2 (3.4%) | 0 |
| Anorexia | 13 (22.4%) | 0 |
| Aspartate/Alanine aminotransferase elevation | 4 (6.9%) | 2 (3.4%) |
| Cholangitis | 1 (1.7%) | 1 (1.7%) |
| Constipation | 3 (5.2%) | 0 |
| Dyspepsia | 1 (1.7%) | 0 |
| Fatigue | 10 (17.2%) | 1 (1.7%) |
| Institutional lung disease | 1 (1.7%) | 0 |
| Leukopenia | 14 (24.1%) | 3 (5.2%) |
| Mucositis oral | 2 (3.4%) | 0 |
| Nausea | 8 (13.8%) | 0 |
| Neutropenia | 15 (25.9%) | 7 (12.1%) |
| Pancreatitis | 1 (1.7%) | 1 (1.7%) |
| Rash maculopapular | 13 (22.4%) | 0 |
| Thrombocytopenia | 5 (8.6%) | 0 |


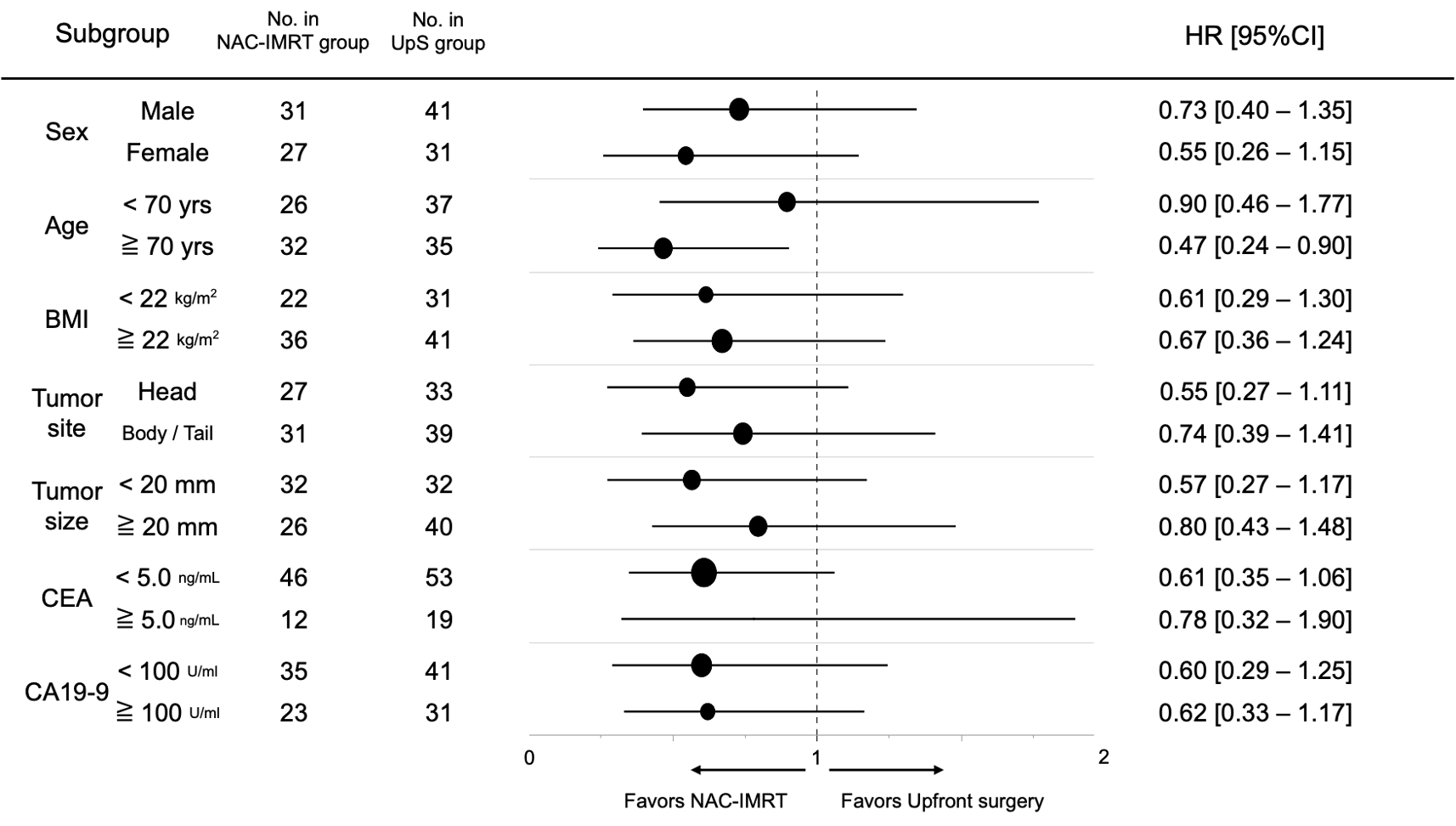


Figure S1. Hazard ratios for the impact of NAC-IMRT on overall survival across subgroups; BMI, body mass index; CA19-9, carbohydrate antigen19-9; CEA, carcinoembryonic antigen; NAC-IMRT, neoadjuvant chemoradiotherapy with using intensity-modulated radiation therapy; UpS, Upfront surgery

****Figure S2. Kaplan–Meier estimates of OS comparing recurrent cases in the As-treated set based on the presence or absence of GnP or mFFX therapy. (A) The entire cohort; (B) subgroup analysis in the NAC-IMRT and UpS groups. In all analyses, no significant differences in OS were observed between groups with and without GnP or mFFX therapy.
